# Use of a Heart Team in Decision-Making for Patients with Complex Coronary Disease at Hospitals in Michigan Prior to Guideline Endorsement

**DOI:** 10.1371/journal.pone.0113241

**Published:** 2014-11-21

**Authors:** Jeffrey T. Bruckel, Hitinder S. Gurm, Milan Seth, Richard L. Prager, Andrea Jensen, Brahmajee K. Nallamothu

**Affiliations:** 1 Massachusetts General Hospital, Edward P. Lawrence Center for Quality and Safety, Boston, MA, United States of America; 2 University of Michigan Health System, Division of Cardiovascular Medicine, Ann Arbor, MI, United States of America, Blue Cross Blue Shield of Michigan Cardiovascular Consortium, Ann Arbor, MI, United States of America, University of Michigan Health System, Ann Arbor, MI, United States of America; 3 University of Michigan Health System,Department of Cardiac Surgery, Ann Arbor, MI, United States of America, Michigan Society of Thoracic and Cardiovascular Surgeons Quality Collaborative, Ann Arbor, MI, United States of America; 4 Blue Cross Blue Shield of Michigan Cardiovascular Consortium, Ann Arbor, MI, United States of America; 5 University of Michigan Health System, Division of Cardiovascular Medicine, Ann Arbor, MI, United States of America, Ann Arbor VA Center for Clinical Management and Research, Ann Arbor, MI, United States of America; Kaohsiung Chang Gung Memorial Hospital, Taiwan

## Abstract

**Background:**

Revascularization decisions can profoundly impact patient survival, quality of life, and procedural risk. Although use of Heart Teams to make revascularization decisions is growing, data on their implementation in the real-world are limited. Our objective was to assess the prevalence of Heart Teams and their association with collaboration in routine practice.

**Methods:**

A survey of cardiologists and cardiac surgeons at 31 hospitals in Michigan was performed in May, 2011 – prior to the recommendation for using Heart Teams in national guidelines. This survey included all percutaneous coronary intervention-performing hospitals in Michigan participating in the Blue Cross/Blue Shield of Michigan Cardiovascular Consortium and Michigan Society of Thoracic and Cardiovascular Surgeons Quality Collaborative. It targeted both the use of Heart Teams and multidisciplinary Case Conferences.

**Results:**

There were 53 physician survey respondents from 27 hospitals with 4 hospitals not responding. Among respondents, 11 (40.7%) hospitals reported no Heart Teams or Case Conferences while 7 (25.9%) hospitals reported either a Heart Team or Case Conference. However, there was disagreement about the presence of a Heart Team at seven hospitals, and about Case Conferences at nine hospitals. Hospitals with definite Heart Teams reported significantly greater levels of collaboration between cardiologists and cardiac surgeons.

**Conclusion:**

The overall presence of Heart Teams prior to their recommendation in national guidelines was limited. Even among hospitals with a potential Heart Team, there was substantial disagreement between respondents about their presence. Further refinement of the definition of a Heart Team and measures of successful implementation are needed.

## Introduction

The Heart Team is a new concept in decision-making between cardiologists and surgeons for patients with complex coronary disease. This concept has become more widespread after publication of the influential SYNTAX trial.[1] The decision to enroll a patient in the trial was based on a consensus for suitability for either method of revascularization (coronary artery bypass grafting, CABG, or percutaneous coronary intervention, PCI) between an interventional cardiologist and a cardiac surgeon. This process was initially designed to streamline inclusion criteria and provide for a broader range of patients than prior trials of revascularization. Since publication of the SYNTAX trial, however, the concept of a Heart Team has been more broadly expanded to routine clinical care. Use of a Heart Team is now a Class I recommendation in management of patients with complex coronary disease in guidelines issued by American and European professional organizations.[2]

Although enthusiasm for the Heart Team concept is growing and theoretically well founded, this concept is still in its developing stages and there are almost no empirical data on its use in the real-world. For instance, we know very little about its institutional prevalence, the structure of Heart Teams, and their relationship to collaboration among cardiac surgeons and cardiologists in routine practice. One of the first reports of successful program implementation was recently published demonstrating feasibility of introducing the process into routine care, but this was a single-center experience and did not reflect the challenges many different institutions may be experiencing.[3] As such, recent reviews have emphasized the need for more on the use of Heart Teams.[4] Accordingly, we report the findings of a statewide survey of hospitals performing percutaneous coronary intervention (PCI) in Michigan, designed specifically to assess the prevalence and characteristics of Heart Teams at revascularization-capable hospitals throughout the state. This survey was performed prior to the formal recommendation of Heart Teams in clinical practice guidelines. It is crucial to understand the early experience with Heart Teams prior to designing more targeted and more comprehensive programs.

## Methods

The Blue Cross/Blue Shield of Michigan Cardiovascular Consortium (BMC2) and the Michigan Society of Thoracic and Cardiovascular Surgeons (MSTCVS) Quality Collaborative maintain statewide registries for all patients in the State of Michigan undergoing PCI and adult cardiac surgery.[5], [6] These registries includes 31 hospitals where both CABG and PCI are performed; these institutions with full revascularization capabilities made up the sample of where surveys were conducted. The goal of the survey was to identify the prevalence of Heart Team activities, and the characteristics of decision-making for patients with unprotected left main coronary artery disease (LM-CAD) and/or multi-vessel coronary artery disease (MV-CAD) at those institutions. The BMC2 is a physician-run quality improvement collaborative that is supported by but independent of the funding agency, Blue Cross Blue Shield of Michigan. A physician advisory committee is responsible for setting the quality goals and developing quality improvement efforts without any input from or sharing of data with the study sponsor. At the time of the survey, there were 31 hospitals participating in the registry. Each participating institution has identified a physician quality champion who is responsible for local quality improvement and for collaborating with other institutions and the registry. MSCTVS has a similarly run quality improvement collaborative with a focus on cardiac surgery.

The University of Michigan Institutional Review Board has waived the need for approval of studies based on the data collected by the BMC2 registry. Survey responses were collected directly by BMC2 and were de-identified prior to analysis. Individual informed consent was not requested from patients for use of their records as clinical data are submitted directly to the BMC2 registry in a de-identified fashion by participating institutions. Analyses of de-identified patient records were performed by BMC2 staff.

### Survey

The survey was performed in May, 2011, prior to the endorsement of recommendations for Heart Teams in the management of complex coronary disease. The survey targeted physician champions at each institution; it consisted of approximately equal numbers of cardiologists and cardiothoracic surgeons. The physician quality champions were internally selected representatives from each of the participating institutions. The cardiology and cardiac surgery departments at each institution were equally represented. The physician quality champions were the group primarily targeted by the survey, but in some circumstances other participating surgeons and cardiologists completed the surveys.

The survey itself consisted of fourteen questions with binary and scale responses dealing with various topics surrounding the use of teams, participation in Case Conferences, and communication and cooperation between surgeons and cardiologists prior to deciding on revascularization strategies. Collaboration was assessed by asking for the frequency of compliance (divided into four quartiles), and by subjective report (assessed using a five-point Likert-type scale with 1 being “abysmal” and 5 being “best possible”). We explicitly asked for the presence of a Heart Team or multidisciplinary Case Conference that involved both cardiac surgeons and interventional cardiologists. The survey language is in Survey S1.

### Definition of Heart Team

The ACCF/AHA Guidelines define a Heart Team as a multi-disciplinary team composed of an interventional cardiologist and a cardiac surgeon that reviews the patient's medical condition (in cases of unprotected left main and complex CAD) and coronary anatomy, agrees that PCI or CABG is technically feasible and reasonable, and discusses these options with the patient before treatment is selected.[2] We supplied the formal definition of a Heart Team in the survey instructions, but did not make any more specific statements about the structure or makeup of a team (for example, discussion of cases could occur at a scheduled meeting, or an ad hoc basis). The formal definition supplied used the following language: “For patients with stable CAD and multi-vessel or LM disease, all relevant data should be reviewed by a clinical/non-invasive cardiologist, a cardiac surgeon, and an interventional cardiologist (Heart Team) to determine the optimal therapeutic approach including OMT, PCI or CABG.” Case conferences were defined as a “regular combined case conference where the best treatment strategy for patients with MV-CAD or LM-CAD is discussed”.

We used survey results to group institutions into 3 categories: definite, possible, or no Heart Team at their institution. Definite Heart Team was defined as institutions where there was complete agreement between all survey respondents about the presence of either a Heart Team or multidisciplinary Case Conference. Possible Heart Team was defined as institutions where there was discordance of responses between respondents about the existence of a Heart Team or Case Conference. No Heart Team was defined as institutions where there was complete agreement between all survey respondents that no Heart Team or Case Conference existed.

### Statistical Analysis

Survey responses were analyzed using univariate comparisons. Continuous variables were analyzed using means and t-tests. Categorical variables were analyzed using χ^2^ tests, or using Fisher's Exact test (if there were a small number of responses in some categories). Agreement was assessed as the percent of hospitals with complete agreement between all respondents about Heart Team (or case conference) status. Hospitals with only a single respondent were excluded from this analysis (nine hospitals). Statistical analysis was performed using IBM SPSS statistical package, Version 21, 2012, and R ×64 Version 3.0.1. Statistical significance was determined at the 0.05 level.

## Results

There were 53 survey respondents from 27 hospitals who responded to the survey, out of the 31 hospitals participating in BMC2 at the time of the survey (87.1% hospital response rate). The respondents included 31 cardiac surgeons, 21 interventional cardiologists and 1 noninvasive cardiologist. There was a wide range of hospital sizes, and a mix of community and teaching hospitals (Table 1). Nine hospitals had only a single survey respondent. There were 18 hospitals with more than one survey respondent, constituting 44 survey respondents. The number of respondents per facility ranged from one to five. This usually consisted of at least one surgeon and one cardiologist physician champion, except for one hospital where two surgeons and no cardiologists completed the survey.

**Table 1:** Hospital Characteristics for Calendar Year 2012.

	Heart Team		Heart Team		Heart Team		Non-Heart Team		
	Definite		Possible		Total				
	n	% or ±SD	n	% or ±SD	n	% or ±SD	n	% or ±SD	*p*
**N (hospitals)**	7		9		16		11		*—*
**Hospital PCI volume (Total discharges, 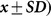 **	1208	±618	831	±576	905	±537	759	±370	*0.35*
**% Teaching Hospitals**	*—*	100%	*—*	100%	*—*	100%	*—*	73%	*0.06*
**% Public Hospitals**	*—*	14.3%	*—*	22.2%	*—*	18.8%	*—*	36.4%	*0.39*
**% Clinical Trial Site**	*—*	28.6%	*—*	33.3%	*—*	31.3%	*—*	36.4%	*1.0*
**% Medicare**	*—*	50.4%	*—*	50.8%	*—*	50.6%	*—*	60.7%	0.02**
**% Managed Care**	*—*	29.3%	*—*	20.0%	*—*	24.1%	*—*	18.6%	*0.62*
**Interventional Cardiologists>1 procedure in 2012 ( 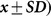 **	18.7	±12.6	13.0	±7.8	14.6	±9.6	13.5	±8.3	*0.72*
**Total PCI Discharges**	8456	*—*	7484	*—*	15940	*—*	8349	*—*	*NA*
Prior CABG	1708	20.2%	1366	18.3%	3074	19.3%	1451	17.4%	<0.001**
STEMI	968	11.4%	1019	13.6%	1987	12.5%	1116	13.4%	*0.18*
Emergent PCI	146	1.7%	126	1.7%	272	1.7%	149	1.8%	*0.76*
Analysis Cohort	5634	66.6%	4973	66.5%	10607	66.5%	5633	67.5%	*—*

*Reported p-values are for the comparison between all Heart Team hospitals (Definite and Possible) versus non-Heart Team hospitals.*

### Prevalence of Heart Teams

Eleven hospitals (40.7%) reported no Heart Team, comprising 16 (30.2%) of survey respondents. Seven hospitals (25.9%) reported a definite Heart Team or Case Conference, comprising 11 (20.8%) of survey respondents. Nine hospitals (33.3%) had a possible Heart Team, comprising 26 (49.1%) of survey respondents.

**Figure 1:**
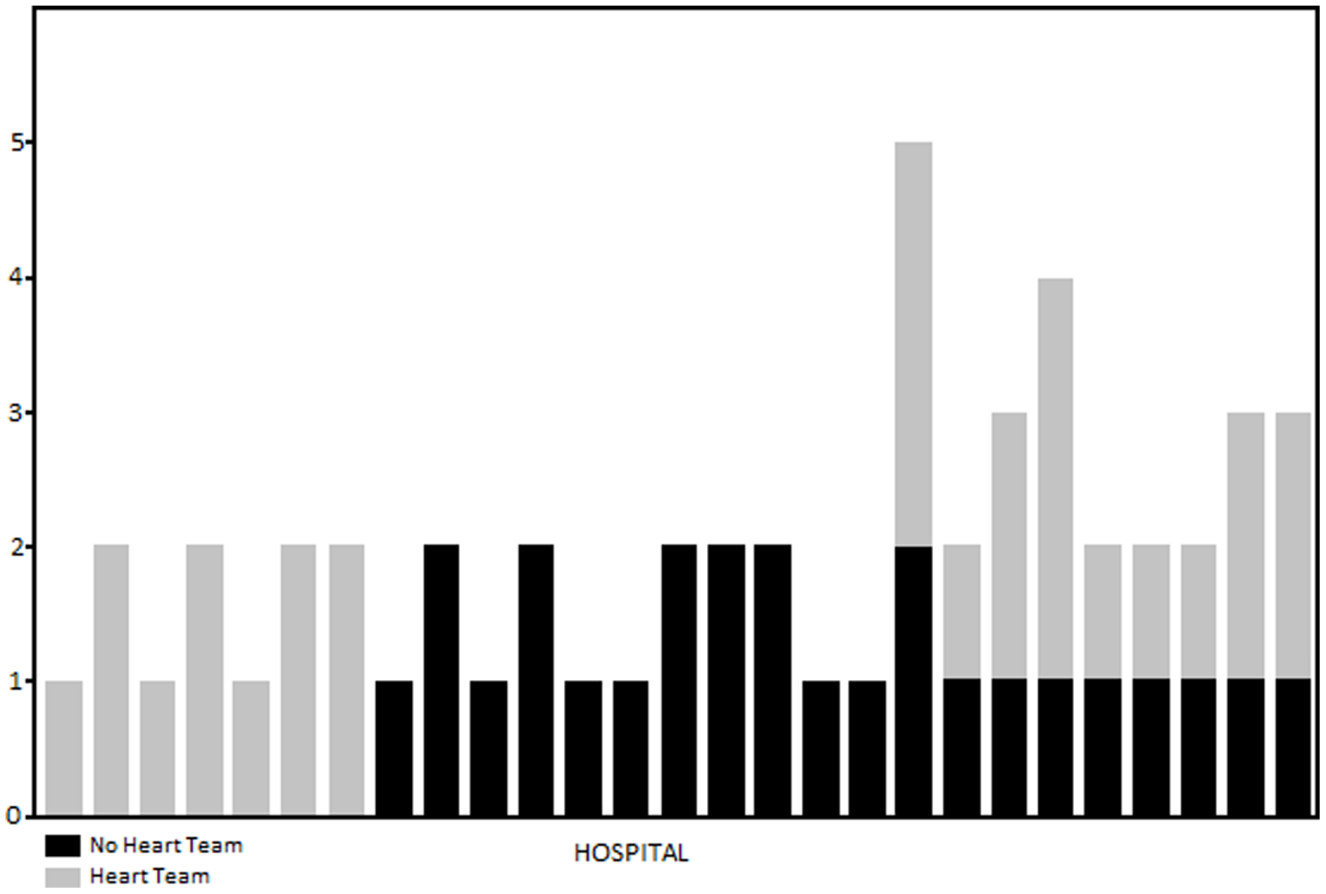
N of survey responses for Heart Team status by hospital.

There was substantial disagreement among survey respondents regarding whether a Heart Team or multidisciplinary Case Conference existed at their institution. There was disagreement about the presence of a Heart Team at seven institutions, and about Case Conferences at nine institutions. These results are highlighted in Figure 1. There was complete agreement on the reported presence of a Heart Team at 61.1% (95% CI 38.6 – 79.7) of hospitals, and complete agreement on the presence of a case conference at 50% (95% CI 29.0 – 71.0) of hospitals. There were no differences in reporting on Heart Teams between specialties *(*p = 0.9 by Mantel-Haenszel-stratified common odds ratio). The institutional and demographic characteristics of hospitals with and without Heart Teams are compared in Table 1. All of the Heart Team group hospitals (definite and possible) were teaching hospitals, compared with 73% of the non-Heart Team hospitals (p = 0.056).

### Collaboration and Communication

**Figure 2:**
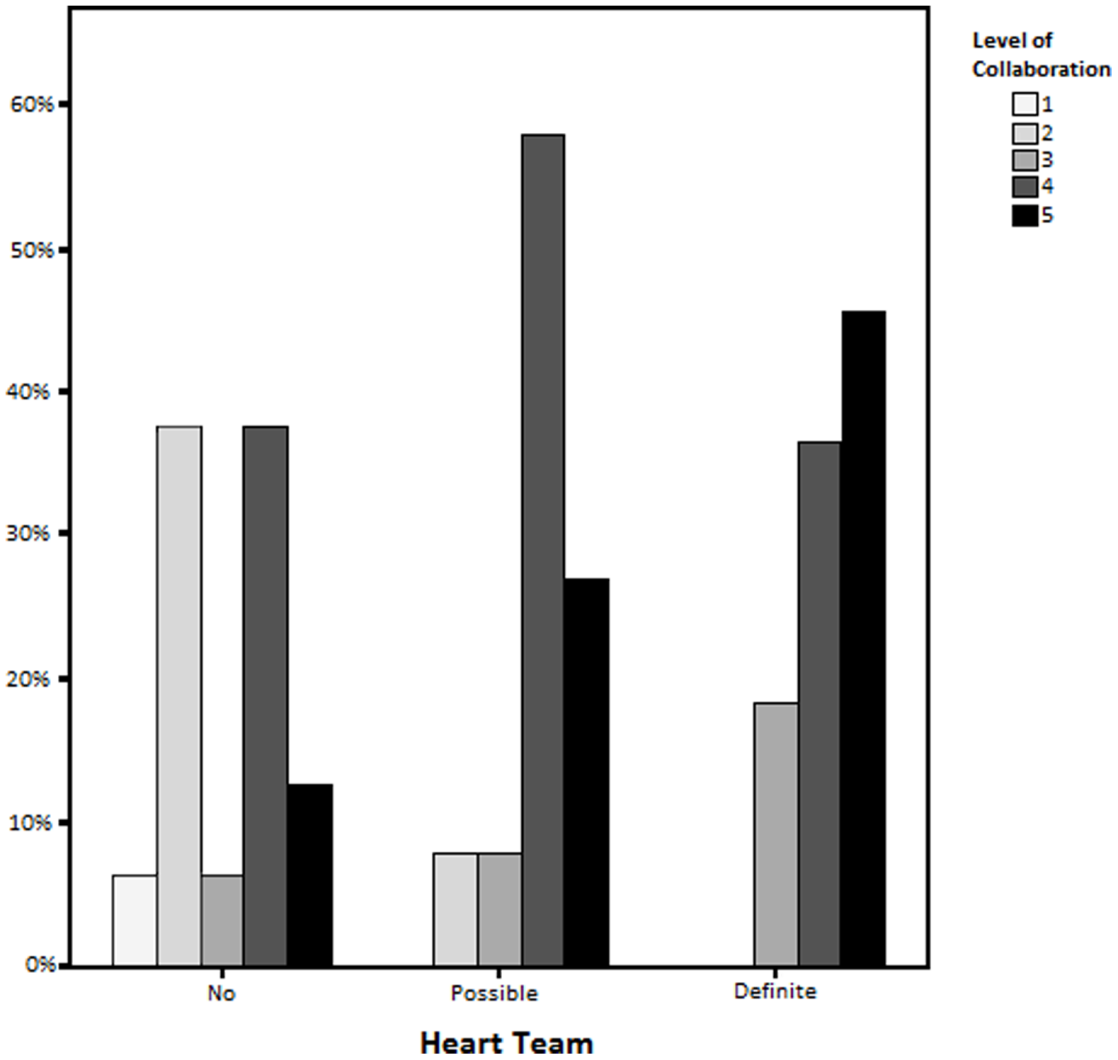
Survey respondent self-reported level of collaboration by Heart Team status.

The subjective level of collaboration between surgeons and cardiologists when coordinating care was assessed. There was significantly more collaboration reported among respondents at institutions with Heart Teams compared with those without (Figure 2). Collaboration was rated as very good or best possible (four or five out of five) at 83.8% of institutions with possible or definite Heart Teams, compared with 50% of respondents from institutions without Heart Teams (*p = 0.017*). There was no difference in the overall level of reported collaboration between cardiologists and surgeons. Communication between surgeons and cardiologists about patients with complex coronary disease was also assessed. There was a greater level of communication about patients undergoing MV-PCI reported by respondents at institutions with Heart Teams compared to those with possible Heart Teams or no Heart Team which was statistically significant (*p = 0.007*). Comparing institutions with Heart Teams to those with a possible or no Heart Team, there was no difference in the reported level of discussion about patients undergoing LM-PCI (p = 0.563), or for patients undergoing MV-CABG or LM-CABG (*p = 0.796* and *p = 0.253*, respectively).

SYNTAX score was used more often by respondents at institutions with definite or possible Heart Teams, compared to those without (*p = 0.035*). There was no difference in SYNTAX score use between institutions by specialty (p = 0.46). These results are shown in Table 2. There was no difference in the utilization of STS scores between respondents from institutions with Heart Teams, or by specialty.

**Table 2:** STS And Syntax Score Use.

			Never	1– 24%	25–49%	50–75%	76–100%	*p*
STS	Definite	n (%)	2 (18.2)	4 (36.4)	1 (9.1)	3 (27.3)	1 (9.1)	*0.46*
	Possible	n (%)	5 (19.2)	5 (19.2)	6 (23.1)	5 (19.2)	5 (19.2)	*—*
	No	n (%)	5 (31.3)	5 (31.3)	2 (12.5)	4 (25)	0 (0)	*—*
SYNTAX	Definite	n (%)	3 (27.3)	4 (36.4)	2 (18.2)	0 (0)	2 (18.2)	0.035*
	Possible	n (%)	7 (26.9)	11 (42.3)	4 (15.4)	2 (7.7)	2 (7.7)	*—*
	No	n (%)	11 (68.8)	2 (12.5)	3 (18.8)	0 (0)	0 (0)	*—*

## Discussion

The survey results from the BMC2/MSTCVS shed for the first time some light into the degree and character of collaboration between surgeons and cardiologists across a broad range of hospitals prior to the recommendation of Heart Teams by national guidelines. The most important findings were the poor baseline use of Heart Teams and significant disagreement between survey respondents about whether a Heart Team or Case Conference exists at their institution. This finding suggests that there have been currently limited opportunities for physicians and hospitals to understand the need for establishing a Heart Team, no clear definition of what activities constitute an active Heart Team, and what criteria will be used to assess whether a Heart Team is present at any given institution. Additionally, our survey evaluates the presence of Heart Teams at diverse and heterogeneous group of hospitals. Although Heart Teams and conferences as venues for collaboration had been in place at larger academic centers prior to guideline implementation, these did not appear to be well-established at smaller institutions at the time of our survey. Implementation of formalized communication structures at community hospitals may be quite different than implementation at an academic center.

The second key finding of the survey was a demonstration of improved subjective collaboration between surgeons and cardiologists at institutions with Case Conferences and Heart Teams. Appropriate treatment decision-making for complex patients requires careful assessment of risks and benefits of each possible treatment. Theoretically this assessment can best be accomplished when hospital culture and processes support a collaborative approach to patient assessment. However, the specific survey items concerning communication and shared patient care activities do not show any definitive difference between institutions Heart Team and/or Case Conference groups, and those without.

Of note, the confusion in identifying Heart Teams is likely to stem from several sources. First, there are no clear guidelines on Heart Team implementation or decision-making for patients with complex coronary disease. In addition, institutions may perceive the makeup and activities of a Heart Team differently. As more publications of implementation reports are made, the makeup and activities of a Heart Team may become more clearly defined. We hope to re-assess this issue in future work that will aim to include site visits and focus group interviews of key leaders at these hospitals.

Our study has the following limitations. The most important limitation is the fact that the survey was conducted prior to the recommendation for Heart Teams in national guidelines. The survey thus represents a baseline assessment of the use of collaborative decision-making models prior to this recommendation. Second, the survey data was based on a small sample size of hospitals. There were typically one or two respondents from each institution, making it difficult to more formally assess communication among all providers. There may also be some selection bias, as those surveyed represented physician quality champions who may possess a more favorable view of the Heart Team model. The survey sample represents a convenience sample, which limits the ability to further generalize the findings outside of Michigan. Additionally, we did not receive responses from all facilities, which could generate some observation bias and loss of statistical power. In order to more thoroughly assess opinions about care coordination at member institutions, a more broad-based survey of all providers should be conducted with a clearer definition of a Heart Team. An assessment using site visits to directly assess the performance of such a team would also be useful. A larger sample size and a more comprehensive group of providers may provide further detail about cooperation within their institutions.

Heart Teams are in their infancy, with the first implementation reports being published in the literature. As implementation of the Heart Team proceeds at institutions, it will become crucial to clearly define the process and outcome measures by which Heart Team activities will be assessed. Heart Teams have the potential to significantly improve care for a large number of patients. The success of a Heart Team at any given institution is dependent on the institutional will to implement such a team, and the working relationships between surgeons and cardiologists. Further study of the Heart Team decision-making process, and reports of implementation of teams will be needed to more clearly define the ideal structure and function of such a team.

## Supporting Information

Data S1 Raw survey data file.
(XLSX)

Survey S1 Original text of the survey as administered to respondents.
(PDF)
