# Supplementary material for: Use of a Heart Team in Decision-Making for Patients with Complex Coronary Disease at Hospitals in Michigan Prior to Guideline Endorsement
Source: PLoS One. 2014 Nov 21;9(11):e113241. doi: 10.1371/journal.pone.0113241 (PMC4240646; doi:10.1371/journal.pone.0113241)
Supplement: Survey S1 — Original text of the survey as administered to respondents. (PDF) [file pone.0113241.s002.pdf]

Heart Team Survey (please fax to: 734-998-6422)

Dear Colleagues,

We are attempting to re-assess the current status of active collaboration between cardiac surgeons and invasive cardiologists at the individual program level. There is an increasing focus on the concept of a "Heart Team" to discuss and consider the most appropriate care of patients with coronary artery disease. For patients with stable CAD and multivessel or LM disease, all relevant data should be reviewed by a clinical/non-invasive cardiologist, a cardiac surgeon, and an interventional cardiologist (Heart Team) to determine the optimal therapeutic approach including OMT, PCI or CABG. *http://www.escvs.com/ESCVS/Home_files/guidelines-revasc-FT.pdf*

The goal of this survey is to define the current practice patterns in Michigan

Hospital Name: ______________________________________City where located: ______________________

Your Specialty: (Circle choice)

Cardiac surgery Interventional cardiology

....

1. Does your hospital host a regular combined case conference where the best treatment strategy for patients with multivessel CAD or LM disease is discussed?

___ No ___ Yes (if yes, frequency)

Weekly once a month twice a month as needed

....

2. What proportion of physicians on staff at your institution attend the conference on a regular basis (at least 80% of the time)? Please indicate for all three.

Cardiac surgeons

0-24% 25-49% 50-75% 76-100%

Interventional cardiologists

0-24% 25-49% 50-75% 76-100%

Non- invasive cardiologists

0-24% 25-49% 50-75% 76-100%

....

3. What proportion of physicians attend this conference even if none of their cases are being presented?

Cardiac surgeons

0-24% 25-49% 50-75% 76-100%

Interventional cardiologists

0-24% 25-49% 50-75% 76-100%

Non- invasive cardiologists

0-24% 25-49% 50-75% 76-100%

4. What proportion of patients undergoing multivessel PCI at your institution are seen by/ discussed with a cardiac surgeon before undergoing PCI?

0-24% 25-49% 50-75% 76-100%

5. What proportion of patients undergoing left main stenting at your institution are seen by/discussed with a cardiac surgeon before undergoing PCI?

0-24% 25-49% 50-75% 76-100%

6. What is the approximate number of left main stents (unprotected) being performed at your institution annually?

0 1-5 6-10 11-15 15-20 > 20

7. What proportion of patients who undergo multivessel CABG at your institution are seen by/discussed with an interventional cardiologist prior to undergoing CABG?

0-24% 25-49% 50-75% 76-100%

8. What proportion of patients who undergo CABG for left main artery disease are seen by/or discussed with an interventional cardiologist prior to undergoing CABG?

0-24% 25-49% 50-75% 76-100%

9. How commonly are procedural risk scores (STS, SYNTAX) used in determination of treatment strategy for patients with multivessel disease or left main artery disease at your institution?

STS

Never 1-24% 25-49% 50-75% 76-100%

SYNTAX

Never 1-24% 25-49% 50-75% 76-100%

10. What is the level of collaboration between cardiac surgeons and interventional cardiologists at your institution? (rate on a scale of 1-5 with 1 being abysmal and 5 being the best possible)

1 2 3 4 5

11. How would you rate the willingness of surgeons to bail out disasters in the catheterization lab? (1 being not willing to 5 being always willing)

1 2 3 4 5

12. How would you rate the willingness of surgeons to take patients in shock for emergency CABG when PCI is not deemed possible or is deemed to be inferior to CABG by the interventional cardiologist? (1 being not willing to 5 being always willing)

1 2 3 4 5

13. How would you rate the willingness of interventional cardiologists to take patients to the cath lab if the surgeon requests a relook after surgery? (1 being not willing to 5 being always willing)

1 2 3 4 5

14. Does your hospital have a "Heart Team" consisting of a clinical/non-invasive cardiologist, cardiac surgeon and an interventional cardiologist to determine optimal care for patients with complex CAD?

______No _____Yes

If yes, please provide some detail of your experience and how it can be used in other institutions.

If no, please detail your expectation of what would be an ideal heart team at your institution.
